# Supplementary material for: Valorization of waste pharmaceutical residues via pyrolysis: Simultaneous production of biochar for Cd2+ removal and high-quality bio-oil/syngas
Source: PLoS One. 2026 Mar 27;21(3):e0346024. doi: 10.1371/journal.pone.0346024 (PMC13028365; doi:10.1371/journal.pone.0346024)
Supplement: Attachment — Submitted filename: Review report on PONE-D-25-58122.docx [file pone.0346024.s001.docx]

**Manuscript No:** PONE-D-25-03329

**Valorization of Waste Pharmaceutical Residues via Pyrolysis: Simultaneous production of biochar for Cd^2+^ removal and high-quality bio-oil/syngas**

This study focuses on production of bio-oil, syngas and biochar. The study has a robust contribution to knowledge as it demonstrates the application of biochar for heavy metal removal., however minor modifications are required to improve it.

1. In Section 2.1 the basic physicochemical properties of the two feedstock biomasses were mentioned but the procedure for the determination of the results in Table 1 was not given. Furthermore, the Table itself is an important part of the results and needs to be moved to the manuscript. The abstract needs to be modified to include the method too.

2. In line 68, page 4, the term pyrolysis gasification is misleading, while pyrolysis occur at the onset of gasification, it does not justify the usage in this context and several places in the manuscript. The authors may need to explain clearly.

3. Please reframe line 121, page six.

4. Please label Figure 1 for easy identification and explanation of the features.

5. The major peaks of Figures 3 and 6 needs to be labelled.

I therefore recommend minor revision before acceptance.
