# Supplementary material for: Valorization of waste pharmaceutical residues via pyrolysis: Simultaneous production of biochar for Cd2+ removal and high-quality bio-oil/syngas
Source: PLoS One. 2026 Mar 27;21(3):e0346024. doi: 10.1371/journal.pone.0346024 (PMC13028365; doi:10.1371/journal.pone.0346024)
Supplement: Attachment — Submitted filename: Response to reviewers.doc [file pone.0346024.s003.doc]

**Response to Reviewers**

**Journal:** PLOS One

**Manuscript ID:** PONE-D-25-58122

**Title:** Valorization of Waste Pharmaceutical Residues via Pyrolysis: Simultaneous production of biochar for Cd^2+^ removal and high-quality bio-oil/syngas

# **Response to Academic Editor Comments**

First of all, the authors would like to express our sincere gratitude to academic editor for providing valuable comments and suggestions on the manuscript all of which we have incorporated into the revised version. All comments will help to improve the quality and clarity of our manuscript. Based on the academic editor comments, we have accordingly revised our manuscript on a point-by-point basis and strictly implemented all the academic editor' comments to revise the manuscript. Revised portions are marked in the manuscript using the different colors (red) in Microsoft Word. The detailed responses to academic editor are given in the following:

**Specific comments:**

**Comments 1:** Please ensure that your manuscript meets PLOS ONE's style requirements, including those for file naming. The PLOS ONE style templates can be found at

https://journals.plos.org/plosone/s/file?id=wjVg/PLOSOne_formatting_sample_main_body.pdf and

<https://journals.plos.org/plosone/s/file?id=ba62/PLOSOne_formatting_sample_title_authors_affiliations.pdf>

Response 1: Thank you for your valuable feedback regarding the formatting of our manuscript. We highly appreciate your attention to detail, which helps ensure our submission adheres to PLOS ONE's established style guidelines. We have carefully reviewed the PLOS ONE formatting templates (main body and title/authors/affiliations) and thoroughly revised our manuscript to address all style requirements, including file naming conventions. Thank you very much.

**Comments 2:** Thank you for stating the following in the Acknowledgments Section of your manuscript: “This research was supported by the Science and Technology Program of Shaanxi Academy of Sciences (Grant No.: 2024k-02), the Science and Technology Program of Xianyang (Grant No.: L2022-QCYZX-NY-006), the Shaanxi Provincial Key R&D Program (Grant No.: 2023‑ZDLNY‑54), and the Shaanxi Provincial Innovation Capacity Support Program (Grant No.: 2023-CX-PT-12).” We note that you have provided funding information that is not currently declared in your Funding Statement. However, funding information should not appear in the Acknowledgments section or other areas of your manuscript. We will only publish funding information present in the Funding Statement section of the online submission form. Please remove any funding-related text from the manuscript and let us know how you would like to update your Funding Statement. Currently, your Funding Statement reads as follows: “This research was supported by the Science and Technology Program of Shaanxi Academy of Sciences (Grant No.: 2024k-02), the Science and Technology Program of Xianyang (Grant No.: L2022-QCYZX-NY-006), the Shaanxi Provincial Key R&D Program (Grant No.: 2023‑ZDLNY‑54), and the Shaanxi Provincial Innovation Capacity Support Program (Grant No.: 2023-CX-PT-12).” Please include your amended statements within your cover letter; we will change the online submission form on your behalf.

**Response 2:** Thank you for your kind reminder. We have removed all funding-related text from the Acknowledgments section of the manuscript as requested.

**Comments 3:** We note that the grant information you provided in the ‘Funding Information’ and ‘Financial Disclosure’ sections do not match. When you resubmit, please ensure that you provide the correct grant numbers for the awards you received for your study in the ‘Funding Information’ section.

**Response 3:** Thank you for your kind reminder. **Please update the online submission form on our behalf using the information below:**

Funding Statement: This research was supported by the Science and Technology Program of Shaanxi Academy of Sciences (2024k-02), the Science and Technology Program of Xianyang (L2022-QCYZX-NY-006), the Province Key Research and Development Program of Shaanxi (2023-ZDLNY-54), and the Science and Technology Department of Shaanxi Province (Grant Nos. 2023-CX-PT-12 and 2025NC-YBXM-122). The funders mentioned in our study had no role in study design, data collection and analysis, decision to publish, or preparation of the manuscript.

**Comments 4:** Thank you for stating the following financial disclosure: “This research was supported by the Science and Technology Program of Shaanxi Academy of Sciences (Grant No.: 2024k-02), the Science and Technology Program of Xianyang (Grant No.: L2022-QCYZX-NY-006), the Shaanxi Provincial Key R&D Program (Grant No.: 2023‑ZDLNY‑54), and the Shaanxi Provincial Innovation Capacity Support Program (Grant No.: 2023-CX-PT-12).” Please state what role the funders took in the study.  If the funders had no role, please state: "The funders had no role in study design, data collection and analysis, decision to publish, or preparation of the manuscript." If this statement is not correct you must amend it as needed. Please include this amended Role of Funder statement in your cover letter; we will change the online submission form on your behalf.

**Response 4:** Thank you for pointing out the mismatch. We have included the amended Role of Funder statement in our cover letter. Please update the online submission form on our behalf using the information below:

The funders mentioned in our study had no role in study design, data collection and analysis, decision to publish, or preparation of the manuscript.

**Comments 5:** PLOS requires an ORCID iD for the corresponding author in Editorial Manager on papers submitted after December 6th, 2016. Please ensure that you have an ORCID iD and that it is validated in Editorial Manager. To do this, go to ‘Update my Information’ (in the upper left-hand corner of the main menu), and click on the Fetch/Validate link next to the ORCID field. This will take you to the ORCID site and allow you to create a new iD or authenticate a pre-existing iD in Editorial Manager.

**Response 5:** Thank you for your reminder regarding the ORCID iD requirement. We have renewed our information in the system.

**Comments 6:** Please include a separate caption for each figure in your manuscript.

**Response 6:** Thank you for your reminder. We have revised the manuscript as requested and included a separate caption for each figure, strictly adhering to PLOS ONE’s formatting guidelines.

**Comments 7:** Please include your tables as part of your main manuscript and remove the individual files. Please note that supplementary tables (should remain/ be uploaded) as separate "supporting information" files.

**Response 7:** Thank you for your guidance. We have revised the manuscript as requested: all non-supplementary tables have been integrated into the main manuscript, and the corresponding individual table files have been removed.

**Comments 8:** If the reviewer comments include a recommendation to cite specific previously published works, please review and evaluate these publications to determine whether they are relevant and should be cited. There is no requirement to cite these works unless the editor has indicated otherwise.

**Response 8:** Thank you for your guidance. We have carefully reviewed and evaluated the specific previously published works recommended in the reviewer comments. Relevant and appropriate studies have been incorporated into the manuscript’s references, while we have not cited those deemed unrelated to the core content of our research, in accordance with your note that citation is not required unless specified by the editor.

**Comments 9:** Please review your reference list to ensure that it is complete and correct. If you have cited papers that have been retracted, please include the rationale for doing so in the manuscript text, or remove these references and replace them with relevant current references. Any changes to the reference list should be mentioned in the rebuttal letter that accompanies your revised manuscript. If you need to cite a retracted article, indicate the article’s retracted status in the References list and also include a citation and full reference for the retraction notice.

**Response 9:** Thank you for your reminder. We have thoroughly reviewed the reference list to ensure its completeness and accuracy. No retracted papers are included in the revised reference list.

# **Response to Reviewer #1 Comments**

**General comments:** The study has some deficiencies and can be accepted for publication after the corrections/additions listed below are completed.

**Response:** We sincerely thank you for allowing us to revise the manuscript, and we also deeply appreciate your kind suggestions and constructive comments on this manuscript. These comments and suggestions could significantly improve the quality of our manuscript. Furthermore, according to your comments and suggestions, the related points you mentioned have been modified. The point-to-point response can be found below.

**Specific comments:**

**Comments 1:** The abstract provides a good overview, but some specific details could be enhanced for better clarity. For instance, mentioning the specific types of 'tuber' and 'herbal' pharmaceutical residues used (e.g., 'Changyanning Pill' and 'Xinsuning capsule' as noted in the materials section) would immediately provide context to readers unfamiliar with these terms. Additionally, while the abstract states that syngas showed 'limited application potential', a brief reason for this limitation would be beneficial, even if elaborated further in the main text.

Response 1: Thank you very much for your professional comments and kind reminders. Based on your suggestions and combined with related literature, we have added specific drug residue sources (tuber-type Xinsuning capsule and herbal-type Changyanning pill) to the abstract and briefly explained at the end of the abstract that the syngas has a low hydrogen content, is mainly pyrolysis off-gas and therefore has a limited application potential. Please refer to Line 16-18 and 27-28 in the “Abstract” of revised manuscript.

**Comments 2:** Adsorption part was not discussed sufficiently in the introduction section of the study. I suggest that this section be improved with the following up-to-date references:

https://doi.org/10.1038/s41598-024-51587-6

https://doi.org/10.1007/s13201-023-02007-z

https://doi.org/10.5004/dwt.2022.28506

**Response 2:** Thank you very much for your critical comments and nice suggestions. Based on your suggestions and combined with related literature, we have added a discussion of the current status of research on the adsorption of heavy metals (especially Cd^2+^) by biochar in the introduction based on three recent publications provided by the reviewers and citing the following:

[DOI:10.1038/s41598-024-51587-6]

[DOI:10.1007/s13201-023-02007-z]

[DOI:10.5004/dwt.2022.28506].

The addition emphasizes the advantages of biochar in heavy metal wastewater treatment and provides a more solid theoretical background for this study. Please refer to Line 104-109 and the references [24] [25] [26] in the revised manuscript. Thank you very much.

**Comments 3:** The introduction effectively highlights the problem of waste pharmaceutical residues in China and the need for high-value utilization. However, it could strengthen the justification for pyrolysis as the chosen method by briefly comparing its advantages over other mentioned methods (e.g., bioconversion, direct combustion) in the context of the specific waste materials studied.

**Response 3:** Thank you very much for your careful comment. In combination with your comment 3, we have improved the logical relationship of the sentence. Following your suggestion, we have added a paragraph to the introduction comparing the advantages and disadvantages of pyrolysis with those of bioconversion and direct combustion in the resourcing of spent pharmaceutical residues, highlighting the comprehensive advantages of pyrolysis in obtaining three-phase products simultaneously: solid, liquid and gas. In addition, we have added the corresponding references. Please refer to Line 94-99 and the references [19] [20] in the revised manuscript. Thank you very much.

**Comments 4:** In the 'Materials' section, the paper mentions that raw materials were collected from 'Shaanxi Momeide Pharmaceutical Co., LTD.'. While this is a good start, specifying the exact nature or form of these residues (e.g., pre-processed waste, raw plant material) would add important context for reproducibility.

**Response 4:** Thank you very much for your critical comments. Admittedly, this is a mistake due to our negligence, and we sincerely apologize for this. According to your comment, it has been added in “Materials”: “Waste drug residues are wet residues generated during the production process of the pharmaceutical plant, which are crushed and sieved after natural air drying, without chemical pretreatment.”. Please refer to Line126-129 in the revised manuscript.

**Comments 5:** The description of the pyrolysis process is clear regarding temperature, time, and atmosphere. However, mentioning the type of pyrolysis (e.g., slow pyrolysis, fast pyrolysis) more explicitly in the main text, beyond just 'oxygen-limited pyrolysis', would align with common terminology in the field.

**Response 5:** Thank you very much for your comment and strict scientific attitude. Meanwhile, unclear expressions have caused unnecessary misunderstandings. According to some reported literature, It has been clearly stated in “2.2 Preparation of biochar, bio-oil, and syngas via pyrolysis” that “In this study, low-temperature slow pyrolysis was employed with a multi-stage program heating protocol: the heating rate was maintained at 5°C·min⁻¹, the pyrolysis temperature was set at 500°C with a 3 h isothermal holding period, and the system was allowed to cool naturally to room temperature after the completion of the reaction.”. Please refer to Line146-149 in the revised manuscript.

**Comments 6:** The FESEM analysis mentions 'obvious honeycomb polyhedral structures' and 'abundant porous structures'. Including quantitative data or a clearer comparative statement about the pore size distribution (beyond just 'larger specific surface area and pore volume' for HB) would strengthen these observations.

**Response 6:** Thank you very much for your insightful comments and kind reminders. A quantitative description of the pore size distribution have been added to “3.1.5. Specific surface area and pore size analysis”, and the BET results are cited for comparison. The observation that HB has a richer mesoporous structure than TB has also been added. Please refer to Line 286-289 in the revised manuscript.

**Comments 7:** For the XRD analysis, the presence of impurities like CaO, KCl, or SiO_2_ is mentioned. It would be helpful to discuss the potential impact of these impurities on the biochar's performance, especially for Cd^2+^ adsorption, or how they might be mitigated in future applications.

**Response 7:** Thank you very much for your constructive comments. A new paragraph has been added to the “XRD” section to discuss that these minerals may enhance the adsorption of Cd^2+^ by mechanisms such as ion exchange and precipitation but may also clog the pores. In the future, the effect of impurities may be reduced by acid washing, etc. In addition, we have added the corresponding references. Please refer to Line 249-257 and the references [32] [33] [34] in the revised manuscript.

# **Response to Reviewer #2 Comments**

**General comments:** This study focuses on production of bio-oil, syngas and biochar. The study has a robust contribution to knowledge as it demonstrates the application of biochar for heavy metal removal., however minor modifications are required to improve it.

**Response:** We sincerely thank you for allowing us to revise the manuscript, and we also deeply appreciate your kind suggestions and constructive comments on this manuscript. These comments and suggestions could significantly improve the quality of our manuscript. Furthermore, according to your comments and suggestions, the related points you mentioned have been modified. The point-to-point response can be found below. If you still have any questions about this manuscript, please let us know and we will not hesitate one minute to revise it with our best efforts. Thank you very much again for your guidance on our work, and your attitude will inspire us to continue our research in the future. Wish you good health and always remain active in your research, and may happiness be with you forever.

**Specific comments:**

**Comments 1:** In Section 2.1 the basic physicochemical properties of the two feedstock biomasses were mentioned but the procedure for the determination of the results in Table 1 was not given. Furthermore, the Table itself is an important part of the results and needs to be moved to the manuscript. The abstract needs to be modified to include the method too.

**Response 1**: Thank you very much for your critical comments and nice suggestions. We would like to explain your comment as follows. (1) A new subsection “Feedstock characterization (Line 135)” has been added in the “Materials”, describing the determination methods of each index in Table 1. Moreover, the relevant references have been added. (2) Table 1 has been replaced at Line 214. (3) A brief description of the methodology has been added to the abstract: “The biochars were characterized by FESEM, BET, XRD and FTIR, and the feedstock physico-chemical properties were measured by common agricultural chemical analysis methods.”. Please refer to Line 135-143, Line 214 and Line 18-20 and the references [27] in the revised manuscript.

**Comments 2:** In line 68, page 4, the term pyrolysis gasification is misleading, while pyrolysis occur at the onset of gasification, it does not justify the usage in this context and several places in the manuscript. The authors may need to explain clearly.

**Response 2:** Thank you very much for your constructive comments. Based on your suggestions, we have checked and standardized the terminology throughout the text and corrected the term “pyrolysis gasification” to “pyrolysis for gas production” to avoid conceptual confusion. Furthermore, in the “preface”, a brief explanation was given for the differences between “pyrolysis” and “pyrolysis for gas production”. Please refer to Line 69-72 and Line 84-87 in the revised manuscript.

**Comments 3:** Please reframe line 121, page six.

**Response 3:** Thank you very much for your professional comments. The original version of the sentence, which reads, “Weigh 20 g of dried waste pharmaceutical residues, and put into a box-type atmosphere furnace with nitrogen pyrolysis throughout the whole process.” has been modified to read, “Twenty grams of dried waste pharmaceutical residues were weighed and placed into a box-type high-temperature furnace with nitrogen protection, where pyrolysis was performed throughout the entire process.” Please refer to Line 145-146 in the revised manuscript.

**Comments 4:** Please label Figure 1 for easy identification and explanation of the features.

**Response 4:** Thank you very much for your constructive comments and reminder. Admittedly, this was an oversight on our side, for which we sincerely apologize. As you suggested, arrows and labels have been added to Figure 1 to label features such as “Honeycomb porous structure” (Figure 1a and Figure 1c), “Flaky tube wall” (Figure 1b and Figure 1d) and “Crushed particles” (Figure 1b and Figure 1d). Please refer to Figure 1 in the revised manuscript.

**Comments 5:** The major peaks of Figures 3 and 6 needs to be labelled.

**Response 5:** Thank you very much for your constructive comments and reminder. Admittedly, this was an oversight on our side, for which we sincerely apologize. Figure 3 (XRD patterns of the prepared materials) has been labeled with the crystal phases corresponding to the major diffraction peaks (e.g., KCl, CaCO_3_, Graphitic-C). Figure 6 (Total Ion Chromatography) has been labeled with major compound names. Please refer to Figures 3 and 6 in the revised manuscript.

**Comments 6:** I therefore recommend minor revision before acceptance.

**Response 6:** Thank you very much for the reviewer's valuable comments and nice suggestions. We have carefully studied your comments and revised this manuscript on a point-by-point basis accordingly, and your suggestions were strictly implemented in the revised manuscript.
